# Overcoming the not-invented-here syndrome in healthcare: The case of German ambulatory physiotherapists’ adoption of digital health innovations

**DOI:** 10.1371/journal.pone.0293550

**Published:** 2023-12-27

**Authors:** Thomas Huynh, Julia Kroh, Carsten Schultz

**Affiliations:** Kiel Institute for Responsible Innovation, Kiel University, Kiel, Schleswig-Holstein, Germany; The Hong Kong Polytechnic University, HONG KONG

## Abstract

Healthcare is characterized by professional, organizational, and institutional boundaries. Digital health innovations can help overcome these boundaries by providing information access to all healthcare professionals. Such innovations emerge from inputs from different health professionals at different positions along the entire care process and have the potential to substantially change the way in which interprofessional tasks are performed among the involved professionals. Consequently, as less empowered professionals, physiotherapists may resist the adoption of digital health innovations in particular if the innovation is dominated by physicians, and thus the *not-invented-here syndrome* may become a major barrier. We aim to examine whether the origin of a digital health innovation affects German physiotherapists’ adoption decision and whether the collaboration quality and physiotherapists’ proactive job crafting behavior may help overcome adoption barriers. We applied a mixed-method sequential design with a qualitative study one in which we interviewed 20 physiotherapists to provide exploratory insights, and a quantitative study two in which we tested our proposed hypotheses with survey data including an experimental vignette from 165 physiotherapists. Physiotherapists adopt digital health innovations developed by their own professional group more likely than digital health innovations developed by physicians. Our results also confirm that physiotherapists’ job crafting behavior and the quality of the collaboration with physicians weaken the resistance against physician-driven innovations. Our study underlines (1) the need to involve allied health professionals as physiotherapists in digital health innovation development, (2) the relevance of interprofessional collaboration in daily practice and, (3) an open mind set of allied health professionals to cope with innovation adoption barriers.

## Introduction

Digital health innovations include information technologies that can fundamentally change healthcare processes [1]. They encourage accessing, sharing, and transmitting of health information between all actors in the healthcare system (e.g., physicians, therapists and patients) and have the potential to overcome information asymmetries and improve the healthcare delivery [2, 3]. For example, a web-based health system that guides patients through their care journey and implements treatment plans in rehabilitation care, including exercise programs in musculoskeletal physiotherapy [4, 5]. Such digital health innovations can enable real-time, location-independent monitoring of patient rehabilitation, support the collection of clinical information, and facilitate timely discussion of rehabilitation care plans among the different health professionals, helping to fill gaps in the care process [2, 6, 7]. Therefore, integrated care, which involves a coordinated and seamless approach to healthcare delivery, can be greatly enhanced by such digital health innovations [8]. However, and because digital health innovations change existing processes in the health value chain [9], their diffusion relies heavily on the adoption by all health professionals along the entire care process [10]. The effectiveness of digital health innovations depends on data exchange and interoperability between different involved health professionals and the incorporation of shared data into clinical decision-making processes. This ensures continuity of care, even as patients move between multiple care providers or facilities [11].

Although these innovations have various benefits for patients and health professionals, recent studies confirm that they often fail to become part of the care routine practice [1, 6, 10]. Previous literature emphasizes that social, organizational and cultural determinants in the adoption of digital health among different health professionals need to be considered but often remain unclear [6, 12–14]. The various types of health professionals (e.g., physicians, nurses and physiotherapists) engage with patients at specific contact points within the care process, and therefore, necessary interactions and collaboration between these different professionals are often very challenging [15, 16]. Because each actor has its knowledge base and individual interests, silos with professional, organizational, and task-related gaps exist [17–19]. Digital health innovations can help overcome these silos but their adoption can also hampered by interprofessional boundaries and conflicting interests between the different health professionals [1, 6]. With this study, we aim to provide a more profound understanding about challenges in the adoption of digital health innovations across interprofessional boundaries and shed light on the under-researched role of the digital innovations’ origin (i.e., peer driven or driven by other actors in the healthcare value chain) in the adoption.

We focus in this study on the German ambulatory orthopedic care sector, which is recognized for organizationally separate and interdependent office-based practices of ambulatory physicians (e.g., orthopedists and general practitioners) and physiotherapists [20]. Various professionals work closely with physicians to provide care. Health professions, like physiotherapists, that are distinct from medicine and nursing are defined as *allied health professionals* [19]. The broad range of noninvasive physical therapies are essential for treating chronic diseases and hence, is an inherent part of numerous clinical guidelines [21]. As the largest group of allied health professions (along with occupational and speech therapists) in Germany and various further countries, physiotherapists are major cornerstones of the ambulatory care sector [20, 22]. Physiotherapists and physicians take different roles in the ambulatory care delivery process. For instance, a patient has to visit in the first place a physician and only with a corresponding medical prescription, the patient is able to obtain physiotherapeutic services which will be reimbursed by the health insurance [22, 23]. In addition, physiotherapists have to align the method and frequency of their treatment based on the given prescription of physicians [20, 24].

The spatial, organizational and task-related gaps hinder necessary cooperation between the involved health professionals. This results in challenging patient-centered treatment and hampers the quality of care [15, 18, 19]. Furthermore, and the core of our study, the interprofessional boundary between physicians and physiotherapists [16] places a potential burden on the successful adoption and widespread diffusion of new digital health innovations [4]. Medical technology companies consider users’ needs for digital health innovations by involving them throughout the design and development process [1]. While the industry is still mainly physician-centric in the development of novel digital tools and technologies [25], physician-driven innovations may bear the risk of being less accepted by allied health professionals. Physiotherapists may perceive such innovations as less beneficial because they perceive that such innovations only meet the needs and expectations of physicians and not those of physiotherapists [1]. We therefore focus on the role which an innovation’s origin plays in the physiotherapists’ innovation adoption.

Making use of the literature on the *not-invented-here syndrome* (NIHS), an innovation origin outside of the individual’s professional group may has a negative impact on the adoption of an innovation [26, 27]. In previous studies, the NIHS has been predominately explained as a source for resistance against new ideas or technologies, which need to cross organizational boundaries and disciplinary and professional domains of expertise [28]. However, NIHS research did not provide a clear understanding of adequate countermeasures to mitigate this individual resistance toward external knowledge [29]. We argue that a high-quality collaboration between physicians and physiotherapists foster perspective taking and therefore are essential to overcome interprofessional boundaries [16]. As such, we propose that the collaboration quality in physiotherapists’ daily work with physicians may help to overcome the NIHS.

To provide a more holistic and profound understanding, we further concentrate on a potential influencing factor on the individual level of physiotherapists. A frequently proposed driver in strengthening the attitude toward change and fostering individuals’ innovation behavior, particularly at work, is known as *job crafting* that results from an inner motivation to innovate [30–32]. Job crafting can be viewed as a specific type of work behavior in which employees initiate changes at work to make their own work more meaningful, engaging, and satisfying [33, 34]. We argue that job crafting represents a possibility to overcome resistances against external knowledge [35], also in form of digital health innovations originating from physicians.

Consequently, this study contributes to the healthcare and innovation management literature by examining both the interprofessional collaboration quality and the individual job crafting behavior as potential moderators in the relationship between the innovation’s origin and the innovation’s adoption. We follow a mixed-method sequential study design by using qualitative interview data (study 1) and quantitative data from a survey with an experimental vignette (study 2; for a similar approach, refer to Huber et al. [36]). As suggested by Harris and Brown [37], we combined qualitative with quantitative data to provide further confirmatory results and to reduce potential biases that may result from a mono-method design [38]. This study contributes to the discussion on adoption challenges of digital health innovations by providing evidence on strategies to increase innovation adoption, particularly across interprofessional boundaries in the fragmented ambulatory care sector.

## Theoretical framework and hypotheses development

The NIHS involves an irrational devaluation or even rejection of external knowledge, even though this knowledge might be beneficial to the particular group or organization. This persistent decision-making error arises as a result of knowledge boundaries between two individuals or interest groups [26, 27]. The NIHS has been predominately explained as individuals’ resistance toward new ideas or technologies in which these individuals were not involved in the creation process [29]. This individual resistance may be more prominent if ideas and technologies need to cross organizational boundaries and additionally, disciplinary and professional domains of expertise [26, 28].

Digital health innovations emerge from health professionals’ and industry’s combined knowledge and expertise, and comprise a wide range of novel tools as e.g., health information systems [39]. Among other factors, the success of the innovation adoption depends on the extent to which new innovations are ‘‘fit for purpose” from the users’ perspective (i.e., health professionals) [40]. The industry, however, focuses mostly on the integration of physicians in innovation processes instead of focusing on the integration of allied health professionals, like physiotherapists, when it comes to new medical tools and technologies [25]. As a result, the adoption of physician-driven digital health innovations, may face the NIHS and thus, be hampered in further health professional groups [4, 41]. Moreover, healthcare is characterized by professional, spatial, and cultural boundaries between particularly physicians and different types of health professionals that accompanying challenges for effective interprofessional teamwork [17]. As shown in previous research, this results in interest conflicts and barriers in the interprofessional knowledge transfer between the different stakeholders in healthcare, hindering the sustainable adoption of digital health innovations [1, 42]. Therefore, it is conceivable that physiotherapists may sense a stronger professional gap toward physicians and in turn, this may cause problems in the adoption of ideas and knowledge originating from physicians [41]. Consequently, physiotherapists may irrationally devaluate physician-driven innovations, as they may believe that these innovations do not match the needs and requirements of their profession due to the missing involvement in the design process [1]. Therefore, we propose the following relationship–

***Hypothesis 1***. *Physiotherapists show a lower adoption rate of digital health innovations that originate from physicians compared to the adoption rate of innovations that originate from physiotherapists*.

### The moderating effect of collaboration quality

Current research suggests that a lack in communication, missing integration of non-physician health professionals in the decision-making processes, and rather strong decision-making power on the physicians’ side are the main sources of intergroup boundaries in healthcare [43]. In contrast, efforts that encourage the perspective taking of physicians can help reduce the boundaries in the perception of the disadvantaged group [28]. A stronger interaction and communication in the daily work may give rise to physiotherapists’ believing they are an integral part of and recognized actor in the ambulatory care sector [24]. In general, a more intense collaboration also strengthens the establishment of a stronger affective and cognitive connection between the different professional groups [44]. A better cooperation, for example, through collaborative treatment planning, creates a greater decision-making authority for physiotherapists, which increases their acceptance of guidelines and practices that purely stem from physicians [28]. An improved flow of information and exchange of expectations between physiotherapists and physicians enables a better mutual understanding of the professional content of their work-related partners and promises to reach a desired and shared clinical outcome [16]. This may result in a higher openness to external ideas and knowledge [43]. Consequently, we understand collaboration quality as a critical moderator of the relationship between innovation origin and innovation adoption as proposed in hypothesis 1. We believe that physiotherapists who collaborate intensively with physicians in daily treatment planning will face lower levels of the NIHS. Accordingly, we hypothesize the following–

***Hypothesis 2*.** *High collaboration quality weakens the negative effect of the NIHS on physiotherapists’ adoption decision and*, *thus*, *leading to a relative increase in the adoption of digital health innovations that originate from physicians*.

### The moderating effect of individual job crafting

Since the NIHS is defined as the individual’s resistance against external knowledge [29], open-minded individuals with higher levels of proactive work behavior may perceive such external knowledge as less threatening [35]. Such proactive form of workplace behavior is also known as *job crafting*, which originates from an intrinsic motivation [31]. An individual who performs intensive job crafting focuses on making changes to certain aspects of their job in order to improve the overall fit between their own personal characteristics, for example, personal (work) goals and the characteristics of the job itself [33, 34]. Job crafting consists of three different behavioral dimensions. First, individuals can proactively improve their *structural job resources* by themselves. They can achieve this through, for example, the full utilization of their capacities by taking over additional tasks to increase their opportunity for professional growth and autonomy [45]. Second, job crafting includes the increase of the quality and/or amount of interactions with others at work (*social job resources*). This comprises the intensity of contact individuals have with colleagues, non-colleagues, and patients for social support, coaching, and feedback [31, 34]. Third, individuals can enhance the cognition and meaning of their profession by crafting more *challenging job demands* to increase their personal growth and job satisfaction [33]. A high degree of job crafting may not only strengthen the ability to innovate [30, 32], but may also weaken professional boundaries as well as related knowledge transfer barriers [31] and thus the NIHS that occurs between physiotherapists and physicians. Individuals who enhance their job resources and who seek further job challenges voluntarily can improve the affective, cognitive, and behavioral attitude toward external initialized changes [35]. Consequently, these individuals may increase their openness beyond the professional boundaries they have set for themselves. As such, job crafting may encourage independent thinking [34] and individuals therefore place less emphasis on social and/or professional boundaries [46]. We therefore derive the following hypothesis–

***Hypothesis 3***. *Individual job crafting behavior weakens the negative effect of the NIHS on physiotherapists’ adoption decision and*, *thus*, *leading to a relative increase in the adoption of digital health innovations that originate from physicians*.

## Study 1 –Qualitative interviews

### Methodology and data

The aim of our qualitative study is to validate and refine our proposed model by semi-structured interview. As a pretest of our interview structure, we conducted two exploratory interviews with physiotherapists in close geographic proximity. This helped us to refine the interview guideline by gaining a deeper understanding of the role of digital health innovations and the professional relationship between ambulatory physiotherapists and physicians [47]. Our initial interview guideline was based on an exploratory desk research on innovation management literature on digital health and on practice-oriented literature on digital health innovation adoption. For the recruitment of the interviewees, we asked German physiotherapy associations for assistance in disseminating information about our study to their members. We selected for the interviews only physiotherapists who have at least three years of professional experience to ensure that the participants are familiar with the German healthcare system. All interviews were conducted personally and lasted between 20 and 45 minutes (see S1 Table for further information about the interviews and interviewees’ characteristics).

We asked in the interviews about barriers in the adoption of digital health innovations in ambulatory care and the perception of the current role of physiotherapists in innovation processes. The interviews were transcribed and subjected to a qualitative content analysis with two independent coders with the MAXQDA software (Version 2020). To ensure the inter-coder reliability, the coding procedure was repeated twice [48]. In case of different data interpretations, we returned to the interview data and discussed the deviations to assemble consensus in the codes [49]. We applied a combination of deductive and inductive coding [50]. First, the initial and exploratory analysis (following an ‘open coding’ principle as suggested by Corbin & Strauss [47]) led to the identification of various codes of physiotherapists’ innovation adoption barriers, which were labeled using respondent-centric terms. In the next step, the number of codes was deductively reduced by classifying the data into theoretical constructs of the hypothesized relationships.

### Results of study 1

We report the results of the qualitative analysis in line with the proposed hypotheses. Exemplary quotes illustrate the findings and are chosen based on their clarity and representativeness related to the overall themes. A particularly prominent comment from the semi-structured interviews underlines the existence of NIHS and emphasizes the opinion of physiotherapists on physician-driven innovations:

“*In my opinion*, *it is essential that we as physiotherapists are involved in the development of innovations […] because the acceptance of innovations we are supposed to use will be low if we were not involved*. *Involvement ensures to include assumptions and to review challenges from both perspectives (physiotherapists and physicians)*. *We can avoid the implementation of less efficient solutions”*. [Physiotherapist 4, translated from German]

This statement emphasizes the strong urge of physiotherapists to be involved and indicates that the acceptance and adoption intention with regard to innovations that solely originate from physicians is low. At the same time, all interviewees expressed the perception that the current degree of integration of physiotherapists in innovation processes is very low. Most of the interviewees highlighted the missing integration of physiotherapists in the innovation development process as the main concern for new digital health technologies in the ambulatory care, which strengthens the presumption of the existence of the NIHS and thus, supports hypothesis 1.

Further, the interviewed physiotherapists emphasize that a lack of interprofessional collaboration and a lack of knowledge transfer with ambulatory physicians negatively affects both, the emergence of promising digital health innovations and the daily business of patient treatment. As such, these interviewees stated that a more frequent collaboration and exchange of experience on a daily base with physicians plays a key role in the further improvement of the ambulatory care sector. This is so, because interviewees emphasized that the effective treatment planning and the development of *“meaningful digital health tools*” rely on the knowledge of both professions. The overall information exchange with physicians was, however, predominantly described as “*very challenging*” due to “*missing consultation possibilities and rare”* or *“non-calls in case of missing information or documents*”. Apart from this, all interviewees described the collaboration in the form of a common treatment planning with physicians as “*very poor”* and *“not sufficient to ensure quality care*.” As a result, the interviews show that physiotherapists consider a daily collaboration as an essential key for the openness toward physicians’ knowledge and thus, as an important prerequisite for the emergence of new promising innovations in general in the ambulatory care sector as emphasized in the following statement:

"*For new health innovations I would like to have a stronger involvement of physiotherapists in innovation processes […]; probably there are already many motivated colleagues*, *but we also need to work more interprofessionally in our daily work*. *When our input is recognized*, *we will be motivated to drive [innovation] together"*. [Physiotherapist 12]

In this context, the interview results emphasize the essential role of interprofessional collaboration quality, i.e., to overcome the proposed NIHS as hypothesized in hypothesis 2. In addition, the interviewees indicated that physiotherapists are merely physicians’ “*executive hand*” with strictly limited job autonomy, which comes about through a lack of integration in decision-making processes and rare consideration of physiotherapists’ expertise in treatment planning. This perceived limited job autonomy was also linked as barrier in physiotherapists’ motivation to explore new solutions and to collaborate with physicians on new digital health innovations. Interviewee 7 expressed it as follows:

"*When we are supposed to try new [digital solutions] and be innovative*, *we should also get paid and have the same authorities as physicians*. *As such*, *the hierarchies should not exist when we want to realize changes together*.*"* [Physiotherapist 7]

The interview results also support the key role of job crafting in physiotherapists’ innovation adoption. The interviewed physiotherapists underline the demanding working conditions and the lack of resources, which limit both the opportunities and the motivation to collaborate and to engage with digital health innovations. Thereby, the interviewees highlight the central role intrinsic motivation plays when it comes to taking over tasks beyond their formal responsibilities, especially in the absence of extrinsic drivers, such as high monetary payoffs. For example, one physiotherapist stated to the question about barriers of engaging with new digital health innovations at work:

"*[…] personal motivation is always important […] there are certainly many motivated therapists out there who are passionate about their work […] However*, *the job is strenuous and is not well paid*. *Without passion*, *many physiotherapists are convinced that doing therapy is more than enough to do in a workday*. *Motivation for innovation is not paid and rewarded extra*.*"* [Physiotherapist 14]

The mentioned self-initiated engagement to undertake further tasks beyond existing responsibilities and (personal) limits without additional payment, matches the definition of the first job crafting dimension, *increasing structural job resources*. Furthermore, increasing structural job resources was linked in the interviews as an essential driver, not only for the digital innovation adoption but also for the joint development of digital health solutions with physicians. This indicates the importance of job crafting behavior to overcome NIHS. In the context of the development of new digital health solutions, the interviewed physiotherapists emphasized as well the importance of personal commitment to encounter challenges in the daily work by proactively confronting and contacting physicians, as the following comment illustrates:

"*Many colleagues do not realize that things would work out better if you*, *from time to time*, *put in a little more effort*. *For example*, *taking more time and actively discussing the treatment with the patient or calling the physicians directly and exchanging experiences […] but this do not result in better conditions when we do not develop new solutions based on the feedback and exchange"*. [Physiotherapist 11]

The commitment to increase professional social interactions with the objective of improving job outcomes matches the second job crafting dimension, *increasing social job resources*. This further supports our assumption that strong job crafting behavior plays an important role in physiotherapists’ adoption decision of digital health innovations, and therefore supports hypothesis 3.

**Fig 1:**
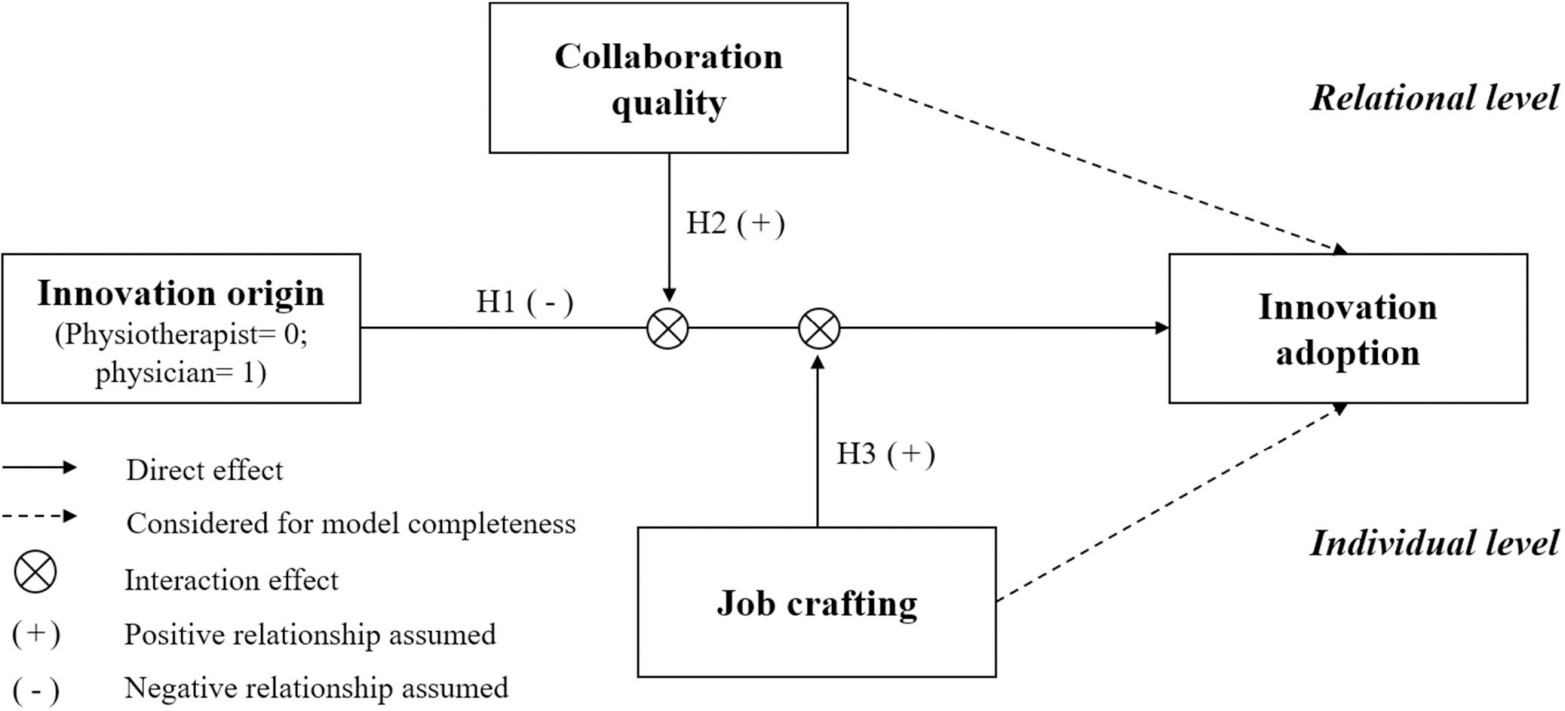
Overall research model.

In sum, the interviews are in line with the theoretical argumentation and provide first qualitative support of our research model and the hypothesized relationships that are summarized in the following Fig 1.

## Study 2 –Quantitative survey

### Methodology and data

To enrich our qualitative findings and to test our hypotheses statistically, we conducted an anonymized online survey in an experimental vignette design with German ambulatory physiotherapists. We used items from previous literature and adjusted them to the current case, if necessary. Our survey questionnaire was pretested with three physiotherapists to increase the validity and reliability of the survey [51]. We recruited participants by placing two announcements in a German professional magazine for physiotherapists, including the link to the survey. We ensured data privacy policy and ethic approval by not including sensible data on the physiotherapists. In total, 229 physiotherapists started the survey but due to incomplete responses, only responses of 165 were used in our study. Double data entry was not possible through technical settings.

### Measures and vignette

We introduced a vignette-based scenario to manipulate the independent variable *innovation origin*. The vignette promised to direct the participants’ attention to specific elements of complex processes, such as the role of the innovation origin on physiotherapists’ innovation adoption [52]. With the vignette, we manipulated the innovation origin of a digital health innovation for information sharing. All participants received a vignette in form of a description of a new digital health information system with which physiotherapists and physicians could provide information and personalized exercises to their patients to facilitate the treatment at home and information exchange (see S1 Fig for full description of the vignette). One half of the participants received the vignette stating that an ambulatory physician designed the digital health innovation for physiotherapists. The other half of the participants received the same description, except that it now stated that the innovation was developed by a physiotherapist. The vignette was displayed in the survey right before the innovation adoption measures to ensure that the participants recognized it sufficiently. We used a dummy variable for innovation origin in our model, with value “0” representing a digital health innovation developed by physiotherapists, and “1” indicating an innovation developed by physicians.

The first moderating variable *collaboration quality* was measured with four items. We asked the participants to rate the perceived degree of co-decision making in the treatment planning (two items) and the communication quality (two items) in the daily work with ambulatory physicians (Lester et al. [44]; sample item: "I work together with ambulatory orthopedists to solve problems and make decisions in the alignment of the treatment planning for patients.”). The second moderating variable *job crafting* was measured with 14 items from job crafting literature (Tims et al. [33]; sample item: “I try to develop my capabilities at work.”). The dependent variable *innovation adoption* was measured based on the established technology acceptance model of Davis and Venkatesh ([53]; sample item: “Assuming I had access to the new digital application, I intend to use it.”). The participants rated in six items the *perceived usefulness*, the *perceived ease of use*, and the *personal intention to use* the digital health innovation that was presented in the vignette. All answer categories of the abovementioned variables ranged on a 5-point scale, from 1 = “strongly disagree” 5 = “strongly agree”.

We performed exploratory factor analyses (EFA) based on principle component analysis to estimate all of the study’s constructs. Factors that had an eigenvalue greater than one were retained [54]. To evaluate our constructs’ reliability, we calculated Cronbach’s alpha for each of the constructs and ensured that all the values were above the recommended minimum value of 0.70 and explained more than 50% of their items’ variance [55]. The EFA revealed that all items of the dependent variable *innovation adoption* and of both moderator variables *job crafting* and *collaboration quality* load respectively on one factor. In addition, one item of the job crafting construct was omitted in accordance with the EFA results (see S2 Table for details on the survey and all measures included).

We further calculated variance inflation factors (VIF) and Pearson’s correlation coefficients to ensure that multicollinearity is not a problem [55]. We mean‐centered (z‐scores) both moderating variables *job crafting* and *collaboration quality* before calculating the interaction terms to mitigate any multicollinearity problems [56]. All VIF values of the theorized test models were less than 2.9 and, thus, under the recommended threshold of 3.3, suggesting that multicollinearity was not a notable problem [55].

To reduce the likelihood of unobserved heterogeneity, our models control for *age*, *gender*, *education*, *size of practice*, and *regional population density*. We included these variables, as they are possible confounders that may affect participants’ intention to use a new technology-related health innovation in their health practice commonly applied in other previous studies [6, 15]. We split *age* into three separate binary control variables (*age under 40*, *age over 40*, and *age over 55)*.

### Results of study 2

Table 1 summarizes the descriptive statistics and includes the means, standard deviations, and correlations among the variables. Of those who completed the questionnaire, the majority was men (56.36%) who had an age of 40–55 years (52.12%). On average, 55.15% of the participants had no academic degree and had been working in the field of physiotherapy for 20.68 years. The participants’ practice teams consisted in average of 4.63 physiotherapists (see S3 Table for details on survey respondent characteristics).

**Table 1:** Correlations, means, and standard deviations.

Variable	Mean	SD	1	2	3	4	5	6	7	8	9	10
1. Innovation adoption	3.38	1										
2. Innovation origin (1: physician)	.49	.50	-.47**									
3. Collaboration quality	2.62	.72	.15	.08								
4. Job crafting	3.53	.97	.28**	.04	.29**							
5. Age under 40	.34	.47	.39**	.06	.21**	.53**						
6. Age over 40	.52	.50	-.21**	-.05	-.22**	-.32**	-.75**					
7. Age over 55	.14	.35	-.24**	.01	.03	-.27**	-.29**	-.42**				
8. Gender (1: female)	.43	.49	-.16*	.13	-.19*	.06	.07	-.13	.10			
9. Education (1: academic)	.45	.50	.41**	-.06	.16*	.65**	.61**	-.35**	-.33**	-.08		
10. Size of practice (ln)	1.41	.50	.08	-.01	.12	.12	.17*	-.11	-.07	.01	.06	
11. Regional population density (ln)	6.03	1.39	-.11	.06	.17*	.14	.08	.01	-.13	-.04	.10	-.10

N = 165

**p < .01.

*p < .05

To test the three proposed hypotheses, we applied ordinary least square regression to analyze our quantitative data using SPSS Statistics (Version 27). Table 2 summarizes the regression results. To further reduce the likelihood of multicollinearity, we calculated both interaction effects separately from each other [55]. But we considered for the model completeness also both moderators at the same time to determine the impact of each with the other in a full model (Model 5). We calculated Cohen’s effect size (f^2^) to quantify whether the model effects are weak (f^2^>.02), moderate (f^2^>.15), or strong (f^2^>.35) [57]. Model 1 (R^2^ = 0.25; f^2^ = 0.33) contains the control variables only. The results show that all social-demographic characteristics of physiotherapists (i.e., age and education) and the regional population density have a statistically significant effect on the innovation adoption. As such, higher education has a positive effect on innovation adoption and higher age has a negative effect on innovation adoption. This is consistent with the literature on individuals’ adoption decisions regarding technology-related health innovations [15]. Subsequent, Model 2 (R^2^ = 0.45, f^2^ = 0.66) analyzes the relationship between innovation origin and innovation adoption, thereby supporting hypothesis 1 (*ß* = -0.93, *p* = 0.00). Physiotherapists prefer digital health innovations from their own professional group over innovations from physicians. In Model 3 (R^2^ = 0.49, f^2^ = 0.92), we added the interaction effects of collaboration quality on the relationship between the innovation origin and innovation adoption and thereby find support for hypothesis 2 (*ß* = 0.24, *p* = 0.04). Model 4 (R^2^ = 0.57, f^2^ = 1.32) adds the effect of job crafting on the relationship between innovation origin and innovation adoption, thereby supporting hypothesis 3 (*ß* = 0.72, *p* = 0.00). Finally, our analysis concludes that all models are statistically valid in terms of the conducted F-tests and thus, supports the accuracy of the tested models.

**Table 2:** Results of the regression analysis.

	Innovation adoption				
Predictor variables	Model 1	Model 2	Model 3	Model 4	Model 5
**Control variables**					
Age under 40	reference group	reference group	reference group	reference group	reference group
Age over 40	-.52** (.19)	-.64**(.16)	-.56** (.16)	-.55** (.15)	-.53** (.15)
Age over 55	-.79** (.27)	-.94**(.23)	-.89** (.23)	-.81** (.21)	-83** (.21)
Gender (1: female)	-.30* (.14)	-.19 (.12)	-.13 (.12)	-.08 (.11)	-0.05 (.11)
Education (1: academic)	.46* (.19)	.34* (.16)	.30* (.15)	.38* (.16)	.39* (.16)
Size of practice (ln)	.01 (.14)	-.02 (.12)	-.05 (.12)	-.03 (.11)	-.04 (.11)
Regional population density (ln)	-.12* (.05)	-.10* (.04)	-.11* (.04)	-.09* (.04)	-.10* (.04)
**Independent variables**					
Innovation origin (1: physician)		-.93** (.12)	-.95** (.12)	-.92** (.11)	.93** (.11)
**Moderator variables**					
Collaboration quality			.02 (.11)		.09 (.11)
Job crafting				-.34** (.10)	-.35** (.10)
**Interactions**					
Innovation origin x Collaboration quality			.24* (.12)		.03 (.11)
Innovation origin x Job crafting				.72** (.11)	.70** (.11)
**R** ^**2**^	.25	.45	.49	.57	.59
**Adjusted R** ^**2**^	.22	.43	.45	.55	.56
**Δ R** ^**2**^		.20	.04	.12	.02
**F-value**	8.98**	19.01**	16.33**	24.53**	20.19**

N = 165, unstandardized coefficients and standard errors in parentheses are presented in this table, analyzed in SPSS V27 2020

**p < .01.

*p < .05

### Results of moderation analysis

**Fig 2:**
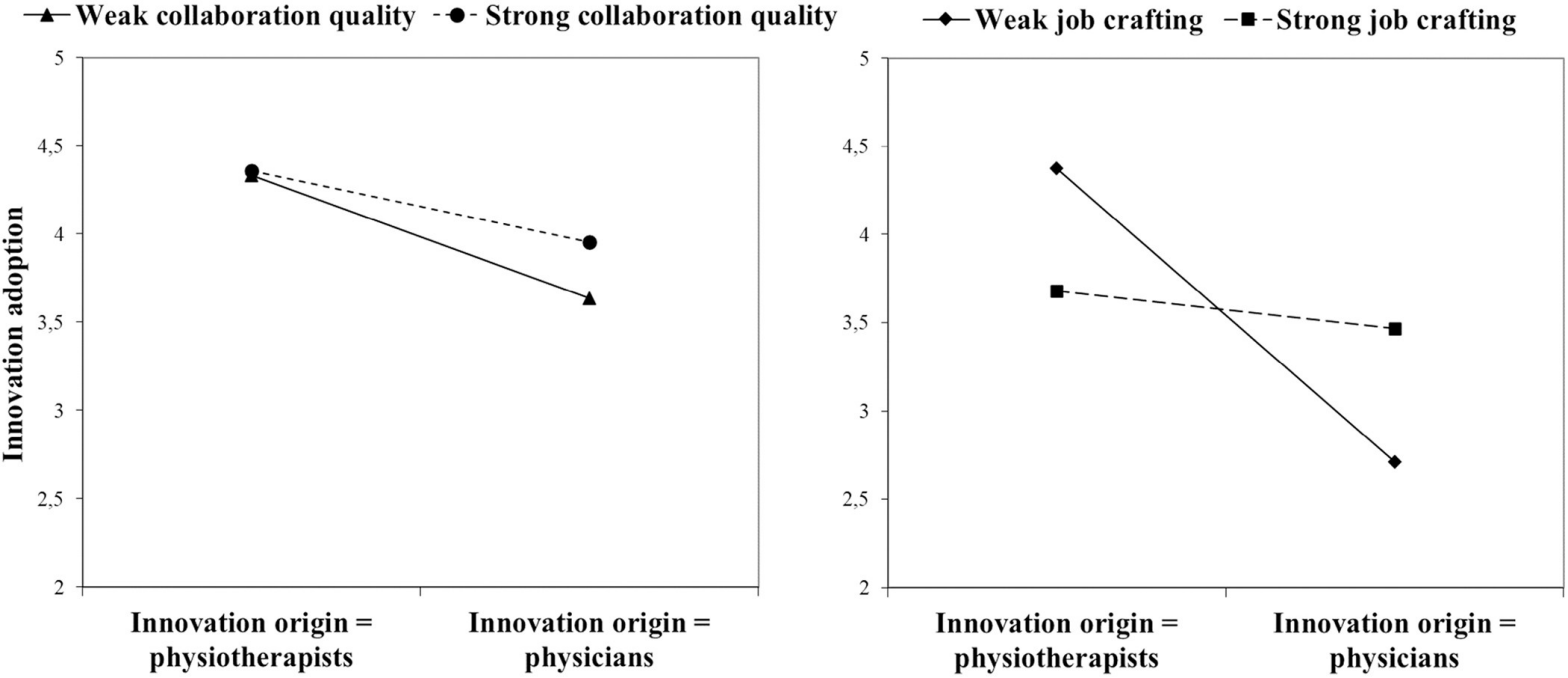
Simple slopes of both significant moderation effects.

To illustrate the regression results of both moderation effects (hypotheses 2 and 3), we further analyzed the simple slopes [56]. The left side of Fig 2 shows the simple slope for the two-way interaction effect of collaboration quality on the relationship between innovation origin and innovation adoption. We focus on high values and low values of the moderator (+/- 1SD), and on the two binary levels of the independent variable. Thereby, the graph illustrates that if the innovation originates from physicians, innovation adoption decreases but that this negative effect is weaken for a higher collaboration quality. If the innovation originates from physiotherapists, we find no influence of collaboration quality on innovation adoption.

The right side of Fig 2 illustrates the two-way interaction effect of job crafting on the relationship between innovation origin and innovation adoption. We focus on low and high levels of job crafting behavior (+/- 1SD). The graph shows the low innovation adoption of innovations originated from physicians. High levels of job crafting avoid this negative effect on physiotherapists’ innovation adoption, in contrast to situations where physiotherapists only possess low levels of job crafting. As such, the graph illustrates that in terms of high levels of job crafting behavior, the innovation’s origin plays a minor role in physiotherapists’ innovation adoption.

## Discussion

By following a mixed-method sequential design with qualitative (study 1) and quantitative data (study 2), this study investigated the role of the innovation origin on physiotherapists’ innovation adoption and its contingencies in the German ambulatory care sector. To the best of our knowledge, this study is the first that applied the NIHS concept to a micro level of healthcare innovation in terms of interprofessional influences on physiotherapists’ innovation adoption. The results underline the essential role of the NIHS in the context of digital health innovation adoption in the ambulatory care setting, where frequently allied health professionals, like physiotherapists and caregivers, must adopt innovations that are dominated by physicians. By emphasizing collaboration quality and job crafting, we explored two underlying mechanisms that can mitigate the negative adoption effect caused by interprofessional boundaries between physicians and physiotherapists.

The interview data from study 1 underlines the relevance of the presumed constructs and relationships derived from theory. As such, the interview findings indicate that the NIHS affects physiotherapists’ innovation adoption. This became apparent when respondents stated that physiotherapists’ intention to adopt digital health solutions in the ambulatory care setting, which are predominantly driven by physicians, will be low. In addition, most of the interviewed physiotherapists perceived the existing lack of integration of their profession in the development process of digital health innovation as a major concern for innovation adoption. We also find statistical support in study 2, where innovations that are developed by physicians have a lower physiotherapists’ innovation adoption rate than digital innovation that are developed by physiotherapists.

Both qualitative and quantitative results highlight the role of the interprofessional collaboration quality in the daily patient treatment to mitigate the NIHS. This is in line with current literature [17, 43] that reveals that lacking communication and lacking integration of non-physician health professionals into daily decision-making processes are the origin of conflicts and strong perceived boundaries toward physicians. This, in turn, weakens the acceptance of decisions and ideas from physicians. Our findings go along with various studies that emphasize that the sustainable adoption and diffusion of digital health innovations requires to overcome the health system fragmentation, as e.g., related interest conflicts and barriers to share knowledge among the involved stakeholders [1, 6, 42]. For example, the study by Karstens et al. [58] found that German general practitioners consider the inefficient collaboration and the related lack of knowledge sharing with physiotherapists, especially in the treatment of complex cases, as main concerns for the implementation of a novel screening tool that is intended to be used in the practices of physicians and therapists. In this context, our study complements existing evidence and emphasizes that interprofessional collaboration in the daily practice plays an essential role to ensure the continuous adoption of digital health and improvement of ambulatory care.

Furthermore, the quantitative and qualitative results also demonstrate that physiotherapists’ job crafting as a type of positive and proactive work behavior is crucial to cope with interprofessional knowledge barriers and, thus, to mitigate the NIHS. Thereby, our findings confirm that job crafting foster the openness toward external knowledge and initiatives beyond the individual’s considered professional boundaries. This goes hand in hand with the study by Demerouti et al.’s [35], which emphasizes that higher job crafting fosters individuals’ openness to externally initiated change, caused by a stronger intention of individuals to act on their own initiative rather than in response to pressure from their immediate professional and social environment. These two moderating variables help us to reveal potential causal effects that explain the observed NIHS.

The interviews of study 1 indicate, that in addition to the conflicts, the perceived power imbalance between physiotherapists and physicians may also play a crucial role in explaining the complex relationship between the innovation origin and innovation adoption [43]. During the interviews, physiotherapists also indicated that they believe they are viewed by physicians as scientifically inferior and as performing only ancillary tasks. Several responding physiotherapists seem to be bringing this personal issue to the forefront rather than exploring new digital solutions and engaging with physicians on new digital health innovations. As emphasized by Antons and Piller [29], resistance toward external knowledge and ideas (i.e., NIHS) can also arise independently from organizational boundaries between groups from different hierarchical levels. In this respect, future studies should investigate perceived power imbalances in decision-making processes in the daily care practice, but also in innovation processes, as further influence factors to gain a deeper understanding of how to cope with the NIHS. The positive role of job crafting in mitigating the NIHS further suggests that the NIHS may stem from limited innovation competences, experiences and as such, from a low innovation related self-efficacy of physiotherapists. Physiotherapists may fear that they are not able to influence the nature and objective of the innovation and hence, may oppose its implementation. Again, further research should more strongly focus on the causal mechanisms of such individual characteristics.

We used a mixed-method design to allow for the exploration of first causal mechanisms [38] and applied a vignette as a quasi-experimental design to limit potential response biases in our survey [59]. Nevertheless, our study has certain limitations. A common-method bias may still affect the findings of our quantitative analysis. Future research should attempt to observe the actual innovation usage and thus should go beyond the focus on the innovation adoption intention. In addition, single-source bias is also conceivable in this study. However, physiotherapists are often self-employed [20] and multi-respondent surveys are rarely possible. Moreover, we cannot exclude the influence of further control variables, mediators and/or moderators that influence physiotherapists’ innovation adoption. We have included other variables in our analysis in study 2. For instance, we tested on the organizational level, the entrepreneurial orientation of the physiotherapy health practice, and on the individual level the personal adherence toward quality assurance guidelines for evidence-based practice. Our conducted analyses revealed no further direct nor moderations effects on physiotherapists’ innovation adoption. However, additional influence factors are conceivable, as for instance, the employment status (self-employed versus employed) or the embedment in integrated healthcare teams. Consequently, more in-depth studies should focus on further antecedents of the revealed innovation adoption barriers across the professional boundaries between physiotherapists and physicians to establish a more profound understanding. Future studies should also consider the perspective of physicians. In addition, it is conceivable that interprofessional barriers may also affect the adoption of digital health innovations across other types of healthcare professionals. For example, Smith and Johnson [19] found, that a lack of interprofessional collaboration hinders the translation of scientific research into evidence-based practice across the different professions in nursing and allied health. Therefore, future research should also empirically examine the potential challenges of innovation adoption across other healthcare disciplines.

## Conclusion and implications

The results of this study suggest that the NIHS has a strong negative influence on physiotherapists’ innovation adoption and show that interprofessional collaboration and job crafting are two mechanisms to overcome the NIHS. We analyzed the ambulatory care sector with physiotherapists as main focus. Our selected case is appropriate since the German ambulatory care is recognized for organizational, and task-related conflicts that leads to inefficiencies in the interprofessional collaboration between involved health professions as physiotherapists and physicians [24, 58]. We expect that the results are transferable to other health professionals, like nurses and/or further providers of curative and therapeutic services, who have a strong influence on the care quality and efficiency, but still play a minor role in innovation development and innovation research.

The study shows that a close collaboration in the daily patient treatment can mitigate personal resistance to adopt physician-driven innovations. Interprofessional relationships are important to ensure a proper development and diffusion of digital health innovations [41]. Physicians and further health professionals as physiotherapists should work on strategies to overcome interprofessional knowledge barriers. Interfaces to improve cross-sectoral cooperation, coordination, as well as information exchange in health service provision therefore become increasingly important [18]. In this context, our findings emphasize the relevance of the management of integrated healthcare processes to minimize interprofessional barriers and information silos. Physicians and other health professionals should collaborate in structured teams to provide care, including, for instance, with regular meetings to share information and coordinate activities. Apart from that, the implementation of shared multidisciplinary guidelines can prevent that each profession or organization follows its own recommendations instead of following a common evidence-based care practice. In the education of physiotherapists and physicians, a multidisciplinary culture could be established early in all disciplines to maximize mutual knowledge transfer.

Since our study was able to confirm the moderating effect of individual job crafting behavior, it is recommendable to encourage physiotherapists to actively and individually design their job. Accordingly, we recommend managers of ambulatory organizations and physiotherapy training centers to encourage a climate for behavioral job crafting strategies. In the case of small, independent one-man practices, the possibilities to motivate physiotherapists are quite limited but could possibly succeed through physiotherapist associations.

Today, physicians are often involved and consulted by firms that develop novel health tools and services. In contrast, further end users like allied health professionals are rarely considered as development partners [25]. Hence, our results indicate that the development of digital health innovations needs to be an interactive process involving a broad set of relevant disciplines, with close relations not only between the industry, clinicians, and academia, but also on the profounder level between the different health professionals [1, 14, 39]. While the relevance of new digital health innovations is further increasing, the high diversity of users also emerges which leads to a variation in requirements [25]. To cope with individual requirements and resistances against novel digital health solutions, it is important to actively integrate physicians and also involved allied health professionals, like physiotherapists, in the development of novel digital health solutions. Because not only physicians but also physiotherapists have direct interactions with patients, they understand patients’ unique needs, challenges, and preferences. Therefore, physiotherapists should be actively involved in the design and testing of new digital health solutions, such as those designed to deliver exercise programs, monitor patient progress, and provide educational resources to patients [7]. Physiotherapists and physicians can come together to form interdisciplinary teams that include other relevant health professionals and technology experts. This team-based approach goes hand in hand with the envisioned concept of integrated care, that collaborative decision-making, in particular during the innovation process, among healthcare professionals can lead to better-designed digital health solutions that address a broader range of diverse needs of patients and thus applicable use cases [7, 8]. The integration of different knowledge bases fosters innovation adoption through a better preference fit with all professional groups to efficiently face existing multifaceted problems in healthcare [6, 40]. In addition, the collaborative development can help increase the adoption of digital health solutions among patients and also foster the patient adherence [25].

Our results are based on the field of digital health innovations, a type of innovation that requires a high level of adaptation of work processes and thus, requires a strong interprofessional collaboration in the innovation development and innovation adoption process [1]. In this context, it is conceivable that complex and analog innovations that aim to change work processes among different types of actors may be influenced by similar effects and thus, our results may be transferable to further innovation types. Physiotherapists are just one example of health professionals that work daily together with physicians. Analogous effects can also be expected for nurses and other providers of curative and therapeutic services. All these health professions have a strong influence on the quality and efficiency of the healthcare system [16]. However, they still play a minor role within innovation processes in practice and also in innovation research. We hope that this study will incite further research to improve this situation.

## Supporting information

S1 Fig Vignette used in survey. (PDF)

S1 Table Characteristics of interviewed physiotherapists. (TIF)

S2 Table Measures, factor loadings, and psychometric properties. (PDF)

S3 Table Sample characteristics of survey participants. (PDF)

S1 Data set (CSV)
