# Supplementary material for: Overcoming the not-invented-here syndrome in healthcare: The case of German ambulatory physiotherapists’ adoption of digital health innovations
Source: PLoS One. 2023 Dec 27;18(12):e0293550. doi: 10.1371/journal.pone.0293550 (PMC10752560; doi:10.1371/journal.pone.0293550)
Supplement: S1 Fig — (PDF) [file pone.0293550.s001.pdf]

Innovation from physicians for physiotherapists - new digital application expands treatment quality

Digitization is making its way into the German healthcare system. Digital innovations such as the "LokalPhysio" application designed by **physicians for physiotherapists**, make it possible to treat patients at any time and from any location. Patients receive easy-to-understand information about their clinical picture and personalized exercises for home in the application from their local physiotherapist or physician. “This could improves the quality of treatment significantly, as patients get a better overall understanding and can now manage their information and recovery much more independently at home…”, says Klaus Müller, an **ambulatory orthopedist and developer** of LokalPhysio. Of course, personal contact is still indispensable: Appointments in person can also be arranged with the physician or therapist in the new application.

Innovation from physiotherapists for physiotherapists - new application expands digital treatment

Digitization is making its way into the German healthcare system. Digital innovations such as the "LokalPhysio" application designed by **physiotherapists for physiotherapists**, make it possible to treat patients at any time and from any location. Patients receive easy-to-understand information about their clinical picture and personalized exercises for home in the application from their local physiotherapist or physician. “This could improves the quality of treatment significantly, as patients get a better overall understanding and can now manage their information and recovery much more independently at home …”, says Klaus Müller, an **ambulatory physiotherapist and developer** of LokalPhysio. Of course, personal contact is still indispensable: Appointments in person can also be arranged with the physician or therapist in the new application.

Note. Texts were translated from German to English.
