# Supplementary figures and images for: Overcoming the not-invented-here syndrome in healthcare: The case of German ambulatory physiotherapists’ adoption of digital health innovations
Source: PLoS One. 2023 Dec 27;18(12):e0293550. doi: 10.1371/journal.pone.0293550 (PMC10752560; doi:10.1371/journal.pone.0293550)

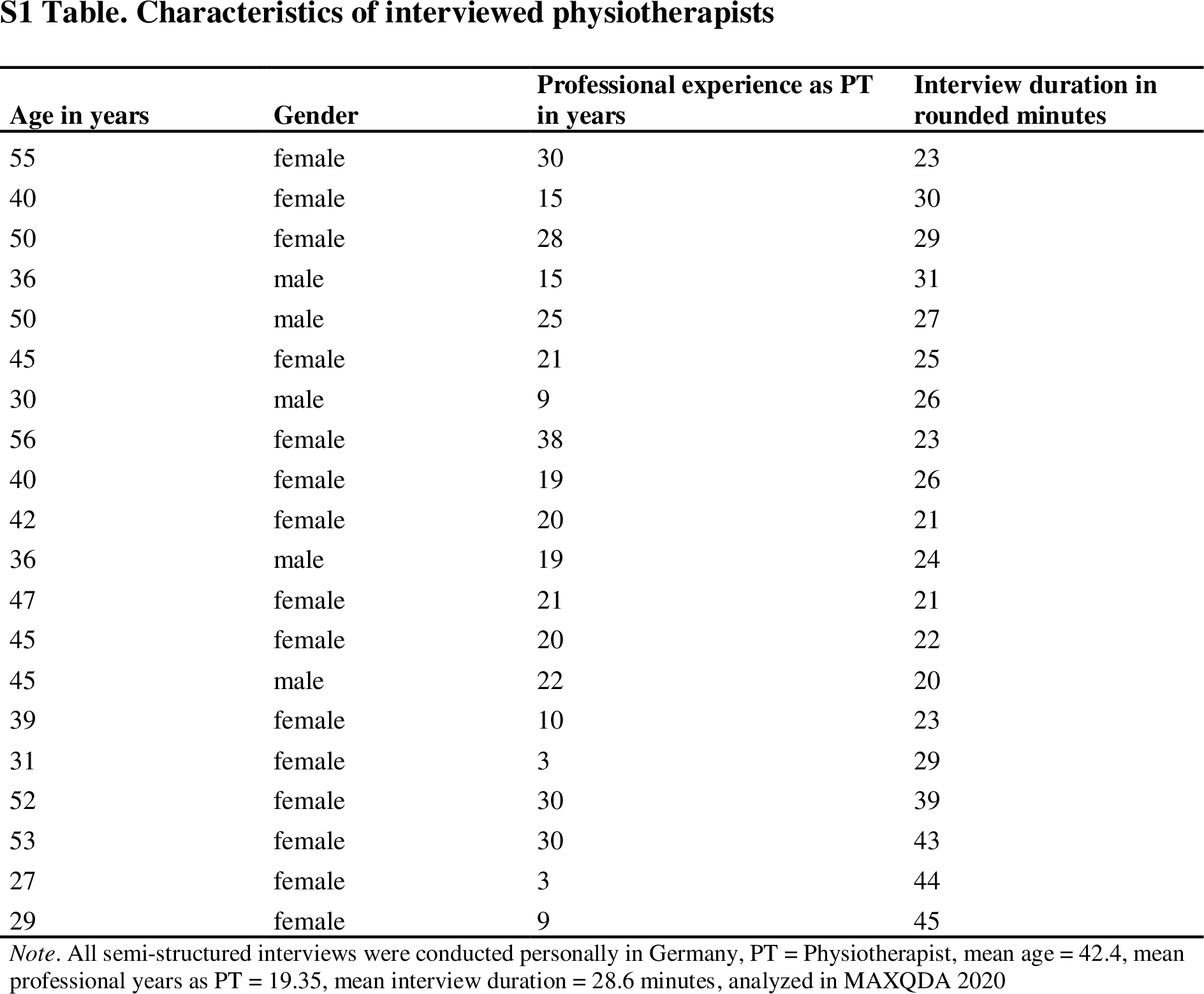

Supplement: S1 Table — (TIF) [file pone.0293550.s002.tif]
