# Supplementary material for: Overcoming the not-invented-here syndrome in healthcare: The case of German ambulatory physiotherapists’ adoption of digital health innovations
Source: PLoS One. 2023 Dec 27;18(12):e0293550. doi: 10.1371/journal.pone.0293550 (PMC10752560; doi:10.1371/journal.pone.0293550)
Supplement: S2 Table — (PDF) [file pone.0293550.s003.pdf]

S2 Table. Measures, corresponding factor loadings, and psychometric properties

Construct and Items	Based on	AVE	α	FL
Scale-based variables (1=strongly disagree, 5=strongly agree)				
**Innovation adoption (Dependent variable)**	Davis & Venkatesh, 1996	.76	.93	
Assuming I had access to the new digital application, I intend to use it [IU1]				.92
Using the new digital application improves the treatment quality [PU1]				.91
Using the new digital application increases the number of patients I can treat [PU2]				.88
Using the new digital application enhances my effectiveness as I can treat more patients in less time [PU3]				.79
I find the new digital application useful at work [PU4]				.93
I find the new digital application easy to use [PE1]				.80
**Collaboration quality (Moderation variable)**	Lester et al., 2002	.61	.79	
I work together with ambulatory general practitioners to solve problems and make decisions in the alignment of the treatment planning for patients [CQ1]				.76
I work together with ambulatory orthopedists to solve problems and make decisions in the alignment of the treatment planning for patients [CQ2]				.79
There are difficulties in the communication and information sharing with ambulatory general practitioners in the treatment of patients [CU1]				.77
There are difficulties in the communication and information sharing with ambulatory orthopedists in the treatment of patients [CU2]				.82
**Job crafting (Moderation variable)**	Tims et al., 2012	.69	.86	
I try to develop my capabilities at work [JCST1]				.89
I try to develop myself professionally [JCST2]				.91
I try to learn new things at work [JCST3]				.87
I make sure that I use my capacities to the fullest [JCST4]				.71
I decide on my own how I do things [JCST5]				.72
I ask people at work to advise me [JCSO1]				.64
I ask whether people are satisfied with my work [JCSO2]	omitted based on EFA results			
I ask people at work for inspiration [JCSO3]				.85
I ask other people outside my organization for feedback on my work [JCSO4]				.84
I ask colleagues in my organization for advice [JCSO5]				.74
When an interesting project comes along, I offer myself proactively as project co-worker [JCCJ1]				.89
If there are new developments, I am one of the first to learn about them and try them out [JCCJ2]				.84
When there is not much to do at work, I think of it as a chance to start new projects [JCCJ3]				.87
I regularly take on extra tasks even though I do not receive extra salary for them [JCCJ4]				.90
I try to make my work more challenging by examining the underlying relationships between aspects of my job [JCCJ5]				.87

Note. N= 165, AVE = average variance extracted, α = Cronbach's alpha, FL = Factor loadings, analyzed in SPSS V27 2020, labels in square brackets correspond to the labels in the original dataset.
