# Supplementary material for: Overcoming the not-invented-here syndrome in healthcare: The case of German ambulatory physiotherapists’ adoption of digital health innovations
Source: PLoS One. 2023 Dec 27;18(12):e0293550. doi: 10.1371/journal.pone.0293550 (PMC10752560; doi:10.1371/journal.pone.0293550)
Supplement: S3 Table — (PDF) [file pone.0293550.s004.pdf]

S3 Table. Sample characteristics of survey participants

Sample characteristics	Participants (N= 165)
**Age groups in years**	
Age under 40	56 (33.94%)
Age over 40	86 (52.12%)
Age over 55	23 (13.94%)
**Gender (1: female)**	
Male	93 (56.36%)
**Education (1: academic)**	
Nonacademic degree	91 (55.15%)
**Size of practice (number of PT)**	
Mean	4.63
**Work experience as PT in years**	
Mean	20.68

Note. PT = Physiotherapist
